# Present and Future Salmonid Cytogenetics

**DOI:** 10.3390/genes11121462

**Published:** 2020-12-06

**Authors:** Muhammet Gaffaroglu, Zuzana Majtánová, Radka Symonová, Šárka Pelikánová, Sevgi Unal, Zdeněk Lajbner, Petr Ráb

**Affiliations:** 1 Department of Molecular Biology and Genetics, Faculty of Science, University of Ahi Evran, Kirsehir 40200, Turkey; mgaffaroglu@yahoo.com; 2 Laboratory of Fish Genetics, Institute of Animal Physiology and Genetics, Czech Academy of Sciences, 27721 Liběchov, Czech Republic; majtanova@iapg.cas.cz (Z.M.); pelikanova@iapg.cas.cz (Š.P.); rab@iapg.cas.cz (P.R.); 3 Department of Bioinformatics, Wissenschaftszentrum Weihenstephan, Technische Universität München, 85354 Freising, Germany; 4 Department of Molecular Biology and Genetics, Faculty of Science, Bartin University, Bartin 74000, Turkey; sunal@bartin.edu.tr; 5 Physics and Biology Unit, Okinawa Institute of Science and Technology, Graduate University, Onna, Okinawa 904 0495, Japan; lajbner@oist.jp

**Keywords:** chromosome banding, cytotaxonomy of trout, FISH, NOR phenotype, rDNA, *Salmo platycephalus*

## Abstract

Salmonids are extremely important economically and scientifically; therefore, dynamic developments in their research have occurred and will continue occurring in the future. At the same time, their complex phylogeny and taxonomy are challenging for traditional approaches in research. Here, we first provide discoveries regarding the hitherto completely unknown cytogenetic characteristics of the Anatolian endemic flathead trout, *Salmo platycephalus*, and summarize the presently known, albeit highly complicated, situation in the genus *Salmo*. Secondly, by outlining future directions of salmonid cytogenomics, we have produced a prototypical virtual karyotype of *Salmo trutta*, the closest relative of *S. platycephalus*. This production is now possible thanks to the high-quality genome assembled to the chromosome level in *S. trutta* via soft-masking, including a direct labelling of repetitive sequences along the chromosome sequence. Repetitive sequences were crucial for traditional fish cytogenetics and hence should also be utilized in fish cytogenomics. As such virtual karyotypes become increasingly available in the very near future, it is necessary to integrate both present and future approaches to maximize their respective benefits. Finally, we show how the presumably repetitive sequences in salmonids can change the understanding of the overall relationship between genome size and G+C content, creating another outstanding question in salmonid cytogenomics waiting to be resolved.

## 1. Introduction

The taxonomic species diversity of peri-Mediterranean and Near East brown trout is still not well understood, and new species are expected to be discovered and/or resurrected from the westernmost tip of the trout distribution area in Morocco [1,2], across the Iberian peninsula [3], Italy, the Balkans (reviewed in Kottelat and Freyhof [4]) and Greece [5]. Similarly, new trout species were recently described in the territory of Turkey in the Mediterranean Sea, Black Sea and Persian Gulf river drainages [6,7,8,9,10,11], presently encompassing 12 species. However, several authors have already recognized the taxonomic diversity of this region’s brown trout ([9,12,13] and references therein). Among these authors, Behnke [14] even erected a new subgenus *Platysalmo* within the genus *Salmo* and described species *P. platycephalus* for morphologically distinct trout found in the Zamanti River in the upper parts of the Seyhan River system in southeastern Turkey. The separate taxonomic status of this species was later confirmed by analyses of mtDNA and nuclear molecular markers, which nested the flathead trout within the Adriatic phylogeographic lineage of the brown *S. trutta* complex and advocated for its separate taxonomic status [13]. However, the exact position of *P. platycephalus* within the Adriatic cluster remained unclear. The researchers concluded that classification of the flathead trout as a genus and/or subgenus of *Salmo* is not supported by their data, although their taxonomic construction is generally accepted in all subsequent studies (e.g., Turan et al. [8]). The flathead trout’s morphological and life history characteristics were addressed by Kara et al. [15,16]. Recently, flathead trout populations are critically endangered by habitat loss and stockings of non-native trout [17].

In spite of numerous cytogenetic studies of the brown trout [18], available data for trout of the peri-Mediterranean as well as the southeastern distribution range remains highly limited (Table 1). In this study, we described for the first time the karyotype and other chromosomal characteristics of the Anatolian endemic flathead trout, *Salmo platycephalus* Behnke, 1968, as revealed by conventional (Ag-impregnation, CMA_3_ fluorescence) and molecular (FISH with 5S and 18S rDNA as well as telomeric probes) techniques. Such a detailed cytogenetic analysis of this species has been missing since the publication of the influential and so far most comprehensive overview of salmonid chromosome evolution [18]. To compare our results with other literature records, we also reviewed available cytotaxonomic data for Eurasian species of the genus *Salmo*. In so doing, we have updated and extensively summarized the present cytogenetics of the salmonid genus *Salmo*.

Current fish cytogenetics has been largely shaped by the huge sequencing effort worldwide, and there are trends to integrate cytogenetics with genomics in fish (e.g., Mazzuchelli et al. [19]; de Oliveira et al. [20]). Salmonids are economically and especially scientifically important [21]; Hence, their genomes have been increasingly sequenced, despite the sizeable obstacles of their genome size [22] and substantial repeats content [23,24] represent particularly to genome assembling. The NCBI/genome currently lists 12 salmonid genomes, of which six species have been assembled to the chromosome level (November 2020). The latest Release 101 of the Ensembl genome browser (August 2020) lists five salmonid species all assembled to the chromosome level [25]. These resources open up new directions for cytogenomic investigations in fish that are particularly relevant for salmonids. Namely, the genome assemblies available in Ensembl can be utilized to produce plots visualizing proportions of repetitive and non-repetitive fractions and their G+C content (GC%) simultaneously with a novel Python tool [26]. Hence, it is now also possible to use this tool for several salmonids and to produce a prototypical virtual karyotype for this group. Actually, the very first plots of *S. salar* are already available by Matoulek et al. [26], in three different resolutions, i.e., different sliding window sizes, (https://github.com/bioinfohk/evangelist_plots). However, *S. salar* belongs to the karyotype category B′ *sensu* Phillips and Ráb [17], i.e., salmonids with 2n = ~60 (54–58) and chromosome arm number NF = 72–74. Hence, *Salmo trutta* Linnaeus, 1758, with a karyotype more similar to *S. platycephalus,* is more desirable for cytogenomic comparisons. The first results of virtual karyotyping of *S. salar* show that the soft-masked genome (i.e., repetitive fraction) appears surprisingly GC-rich (even richer in GC than the non-repetitive fraction [26].

Repetitive sequences that are generally highly important for fish cytogenetics are represented in salmonid genomes in thus far unprecedented proportions of up to 60%, among the highest proportions established for any vertebrate [27,28]. Repetitive sequences in salmonids were recently suggested to have a different relationship between salmonid genome GC% and genome size than that of other teleosts [29]. This is in line with the aforementioned results of virtual karyotyping. Now, thanks to the fast development in fish genomics, new teleost genomes including several salmonid species have become available. Therefore, it is desirable to address this still outstanding question of GC% of salmonid repeatome representing another direction of future research—namely the quantitative approach described in more details as related to fish in this special issue by Borůvková et al. [30]. Hence, we outline future research directions not only of salmonid cytogenetics but of vertebrates’ cytogenetics in general.

## 2. Materials and Methods

### 2.1. Studied Material

Five males and six females of flathead trout were collected by electrofishing in Karagoz Creek, Zamanti River Basin, 38.7350000 N, 36.4864000 E. The individuals were dissected both for direct chromosome preparation in field conditions as well as for other analyses and thus were not deposited in collection as vouchers. Valid Animal Use Protocol was enforced during study in IAPG CAS (No. CZ 02386).

### 2.2. Chromosome Preparation and Staining

Standard procedures for chromosome preparation followed those laid out in Ráb and Roth [31]. Chromosomal preparations from all individuals were stained with conventional Giemsa solution (5%, 10 min) to confirm the number and morphology of their chromosomes. Fluorescent staining with chromomycin A_3_ (CMA_3_) specific for GC-rich regions was applied, counterstained with DAPI, with a higher affinity for AT-rich regions [32]. Silver (Ag-) staining for detection of nucleolar organizer regions (NORs) followed Howell and Black [33]. The sequence of staining followed the protocol of Rábová et al. [34].

### 2.3. Fluorescence In Situ Hybridization (FISH) with Telomeric and rRNA Genes Probes

Probes for FISH experiments were produced by PCR with the primer pairs and thermal cycling conditions according to Komiya and Takemura [35] for 5S rDNA and White et al. [36] for 28S rDNA. The PCR reactions were carried out in a final volume of 25 μL consisting of 100 ng genomic DNA, 12.5 μL PPP master mix, 0.01 mM of each primer and PCR water to complete the volume (all reagents from TopBio, Prague, Czech Republic). Cycling conditions were as follows: (a) 28S: 2 min at 95 °C; 35 cycles of 1 min at 95 °C, 40 s at 55 °C and 2 min at 72 °C; 5 min at 72 °C; (b) 5S rDNA: 5 min at 94 °C; two cycles of 1 min at 95 °C, 30 s at 61 °C, and 45 s at 72 °C; two cycles of 1 min at 95 °C, 30 s at 59 °C and 45 s at 72 °C; two cycles of 1 min at 95 °C, 30 s at 57 °C and 45 s at 72 °C; 25 cycles of 1 min at 95 °C, 30 s at 61 °C and 45 s at 72 °C; 7 min at 72 °C. The amplified fragments were sequenced at the ABI 3700 sequencer prior FISH experiments. Probes were indirectly labelled with biotin-16-dUTP (Roche, Mannheim, Germany) and digoxigenin-11-dUTP (Roche) through PCR reamplification of previously sequenced PCR products. Reamplification was carried out under the same condition as the previous PCR reaction. Labelled PCR products were precipitated. A hybridization mixture was made consisting of hybridization buffer [37], sonicated salmon sperm blocking DNA (15 μg/slide; Sigma-Aldrich, St. Louis, MO, USA) and differently labelled PCR products of both genes. The hybridization and detection procedure were carried out under conditions described by Symonová et al. [37]. The biotin-dUTP-labelled probes were detected by either the Invitrogen CyTM3-Streptavidin (Invitrogen, San Diego, CA, USA; cat. no. 43-4315) or by the FITC-Streptavidin (cat. no. 43-4311). The digoxigenin-dUTP-labelled probes were detected either by the Roche Anti-Digoxogenin-Fluorescein (cat. no. 11207741910) or by the Anti-Digoxigenin-Rhodamin (cat. no. 11207750910). The chromosomes were counterstained with Vectashield/DAPI (1.5 mg/mL) (Vector, Burlingame, CA, USA).

### 2.4. Microscopy and Image Analyses

Chromosomal preparations were examined by an Olympus Provis AX 70 epifluorescence microscope (Olympus, Tokyo, Japan). Images of metaphase chromosomes were recorded with a cooled Olympus DP30BW CCD camera (Olympus, Tokyo, Japan). The IKAROS and ISIS imaging programs (Metasystems, Altlussheim, Germany) were used to analyse grey-scale images. The captured digital images from FISH experiments were pseudocoloured (red for Anti-Digoxigenin-Rhodamine, green for Invitrogen FITC-Streptavidin) and superimposed using Adobe Photoshop software, version CS5. Karyotypes from Giemsa-stained chromosomes were arranged in Ikaros (Metasystems) software. In the case of CMA_3_/DAPI staining, the CMA_3_ signal was inverted into the red channel while the DAPI signal went into the green channel to enhance the contrast between these two signal types. At least 25 metaphases (of the highest possible quality) per individual and method were analysed, some of them sequentially. Chromosomes were classified according to Levan et al. [38], but modified as m = metacentric, st = subtelocentric and a = acrocentric, where st and a chromosomes were scored as uni-armed to calculate the NF value (Nombre Fondamental, number of chromosome arms *sensu* Matthey [39].

### 2.5. Cytogenomic Analyses

First, we reviewed current genomic resources (Ensembl and NCBI/genome) for fish and especially salmonid genome assemblies. Second, we applied the novel Python tool EVANGELIST (= EVAluatioN on GEnome LIST) based on the non-overlapping sliding window to visualize and quantify percentage of repeats and GC% in both repeats and non-repetitive DNA simultaneously, introduced by Matoulek et al. [24]. With this tool, we produced the prototypical virtual karyotype for a salmonid (*S. trutta*). Third, we extracted and manually curated data on genome size and GC% from currently available fish genomes assembled to the chromosome level (the best genome quality) using the NCBI online tool https://www.ncbi.nlm.nih.gov/genome/browse#!/overview/. Finally, we processed these data with R [40] and compared them with our previous results [29].

## 3. Results

### 3.1. Karyotypes and Molecular Cytogenetic Traits

**Figure 1:**
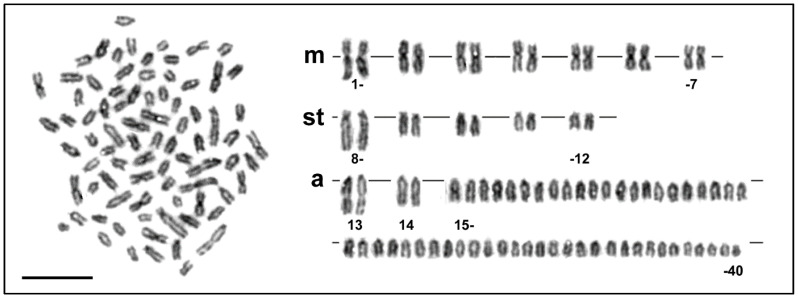
Giemsa stained metaphase plate and the corresponding karyotype of *Salmo platycephalus*. m, metacentric; st, subtelocentric; a, acrocentric chromosomes. Bar equals 10 µm.

**Figure 2:**
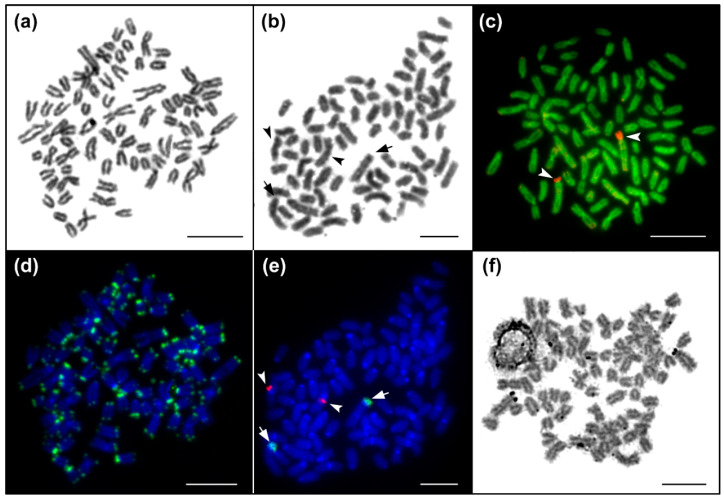
Chromosome analyses of *Salmo platycephalus.* (**a**,**b**) Giemsa-stained metaphases corresponding to (**d**,**e**) panels; (**c**) DAPI/CMA_3_ fluorescence, DAPI stained chromosomes (green), CMA_3_ signals of GC-rich regions (red); (**d**) DAPI stained chromosomes (blue), telomere repeat hybridization signals (green); (**e**) DAPI stained chromosomes (blue), 28S rDNA (green, indicated by arrows), 5S rDNA hybridization signals (red, indicated by arrowheads); (**f**) Ag-NOR impregnation showing the active major rDNA unit corresponding to the 28S rDNA sites. Bar equals 10 µm.

The diploid chromosome number was 2n = 80 and the karyotype was composed of 7 pairs of metacentric, 5 pairs of subtelocentric, 2 pairs of distinctly large acrocentric and 26 pairs of moderate sized acrocentric chromosomes, decreasing gradually in size (Figure 1 and Figure 2a, b). The NF value equalled 96 (Figure 1). DAPI/CMA_3_ fluorescence showed CMA_3_-positive, i.e., highly GC-enriched, signals in p arms of the largest subtelocentric chromosome pair (Figure 2c). FISH with the 28S rDNA probe clearly visualized signals in the same position as CMA_3_, while 5S rDNA sites were located in pericentromeric regions of one middle-sized metacentric chromosome pair (Figure 2e). FISH with the telomeric probe labelled the terminal regions of all chromosomes and did not reveal any interstitial signals (Figure 2d). Finally, Ag-NOR impregnation marked the same CMA_3_ and FISH positive region, i.e., the p arms of the largest sub-telocentric chromosome pairs (Figure 2f).

### 3.2. Virtual Karyotype and Cytogenomics in Salmonids

**Figure 3:**
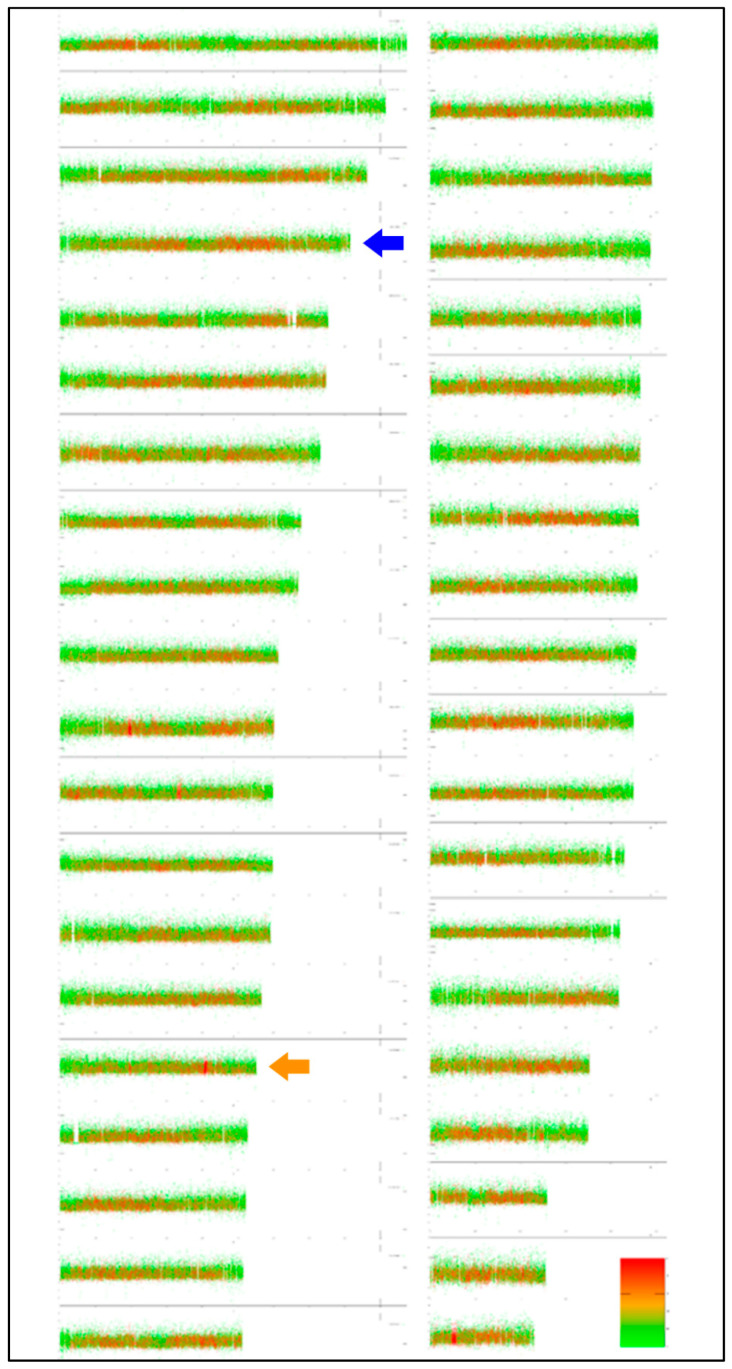
Virtual karyotype of *Salmo trutta* shows the haploid set of size-sorted chromosomes. The colour scale represents the proportion of repetitive (green) and non-repetitive (red) sequences. The *y* axis of each chromosome represents the scale of GC%. The karyotype based on cytogenetics in *S. trutta* enables us to roughly identify only the first three chromosomes according to their size—the largest acrocentric, the largest sub-telocentric and probably the largest metacentric chromosome. According to Ensembl, the 5S rDNA bearing chromosomes are chromosome No. 1 (the main site visualized also by FISH, blue arrow), i.e., the fourth largest chromosome, and chromosome No. 20 (orange arrow), which has a single 5S rDNA sequence.

Thanks to the close relatedness between the *S. platycephalus* analysed here and the species *S. trutta* [41], which is among the best cytogenetically analysed salmonid fishes [18], it is relevant to compare their karyotypes. Utilizing the EVANGELIST Python tool, we produced the first virtual karyotype for the latter species (Figure 3). The virtual karyotype of *S. trutta* was confronted with its cytogenetics-based congeneric karyotypes. Both virtual karyotypes of the genus *Salmo* show a homogenization in GC% along chromosomes and repetitive as well as non-repetitive fractions reaching 50–60%. The comparison with actual karyotypes enabled an assignment of just the three largest chromosomes (Figure 3) at this stage and shows the need to improve the virtual analysis by including an option to visualise the two nuclear ribosomal fractions in the next step. Visualization of 5S rDNA can already be performed in Ensembl, showing that chromosome No. 1 bears the majority of 5S rDNA sequences (Figure 3, labelled with a blue arrowhead). Moreover, one more 5S rDNA site was identified on chromosome No. 20 (Figure 3, orange arrowhead). This site is probably below the detection range of FISH; however, it shows the potential of virtual karyotyping to visualize DNA sequences otherwise hidden for FISH.

**Figure 4:**
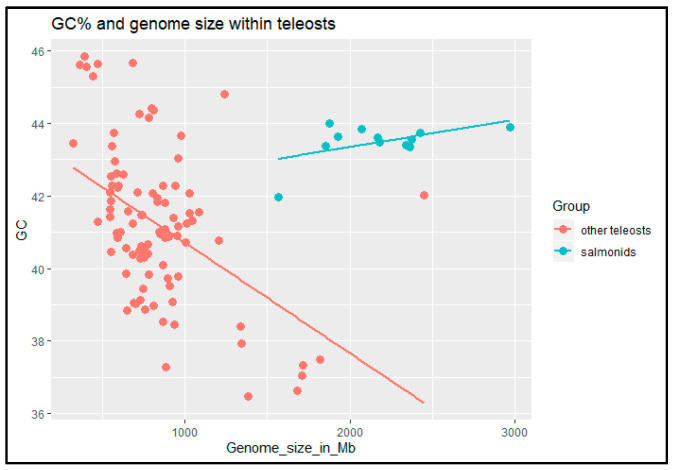
Scatter plot showing the relationship between GC% and genome size in salmonids and other teleosts. Data from https://www.ncbi.nlm.nih.gov/genome/browse#!/overview/.

Finally, we have taken advantage of the increasingly available data on genomic features (GC% and genome size) among teleost fishes with a special focus on salmonids. Including more species than in the previous analysis [29] confirmed earlier results that genome size negatively correlates with the genomic GC% in fish excluding salmonids. Moreover, the results revealed an inverse relationship between these two measures in salmonids in comparison with other teleosts (Figure 4).

## 4. Discussion

The salmonid genus *Salmo* (Linnaeus, 1758) represents freshwater anadromous fishes that are originally widely distributed from the North Atlantic Basin, i.e., Northeastern North America and Europe (including European Arctic) to the upper parts of Amu-Darya R. in Central Asia [42]. The genus contains two sister lineages—a primarily anadromous Atlantic salmon, *S. salar,* and primarily freshwater resident fishes collectively known as brown trout (*Salmo* spp.), although both lineages include numerous anadromous and freshwater populations. However, these salmonids have been introduced and/or stocked outside their native range virtually around the world, mainly as objects of recreational fishery. As a result, several countries report an adverse ecological impact after their introduction [43,44,45,46,47]. Although brown trout are of limited interest in production aquaculture [48] (except the commercially important Atlantic salmon), brown trout have been and still are objects of intense investigations in various types of studies [12]. Similarly, chromosomes of different species and forms of brown trout were already extensively studied by numerous authors by the end of the 19th century (see review of Gas [49]). To compare results of our cytogenetic analysis of flathead trout, we summarized all available chromosome data in brown trout (Table 1). Summing up these studies, we excluded those without reliable locality data, without descriptions of cytogenetic methods used, without the number of individuals used and/or simply reports from other non-referenced sources. In some cases, we reinterpreted specific status of material examined under the name *S. trutta* (i.e., *S. oxianus, S. cenerinus, S. farioides, S. lourosensis, S. peristericus*) since the published locality data clearly pointed to species different from true *S. trutta*. We also included older data based on analyses of anaphase chromosomes from embryo squashes for species/forms not analysed afterwards (i.e., *S. carpio, S. letnica, S. labrax, S. caspius*) but with a sufficient chromosome quality to reliably infer 2n and karyotypes. On the other hand, we are aware that all of these summarized studies had significant flaws. First, none of these studies clearly claimed that examined fishes were deposited in any collection to enable later taxonomic identification of the material analysed [50]. Second, the results of some cytogenetic studies, especially in peri-Mediterranean and central European populations, could certainly have been affected by the stocking of non-autochthonous individuals and their subsequent genetic admixture (e.g., Kohout et al. [51], Leitwein et al. [52]). Nevertheless, our review clearly shows that cytogenetic and/or cytotaxonomic characteristics of flathead trout are nearly or even invariably the same as in other species/forms of brown trout. To explain this conclusion, we further examine in detail the data regarding 2n, karyotype composition and other chromosomal characteristics.

**Table 1:** Review of reported cytogenetic data for members of Palearctic trout of the genus *Salmo.*

Species/Form	Locality	Country	Examined Individuals	2n	Karyotype Composition		NF	Ref.	Notes
					m/sm	st/a			
Aral Sea Basin									
*S. oxianus*	Kyzylsu R. (Amu Darya basin)	KAZ	3	80	18	62	98	[53]	1; 2
*S. oxianus*	Alamedin R. (Chu basin)	KG	5	80	18	62	98	[53]	1; 2
*S. oxianus*	Bech–Tach (Talas basin)	KG	11	80	18	62	98	[53]	1; 2
Balkans and Mediterranean Sea Basin									
*S. carpio*	Garda L.	IT	embryos	80	20	60	100	[54]	1
*S. cenerinus*	Monti Sibillini	IT	57 ^†^	80	14/8	58	102	[55]	2; 5
*S. farioides*	Drosopigi R.	GR	/	80	20	60	100	[56]	1; 2
*S. lourosensis*	Louros R.	GR	/	80	20	60	100	[56]	1; 2
*S. letnica*	Ochrid L.	MK	embryos	80			104	[57]	1
*S. marmoratus*	Socha R.	SI	/	80	22	58	102	[58]	1
*S. marmoratus*	Socha R.	SI	1	80	22	58	102	[59]	1
*S. marmoratus*	Idrijca R.	SI	2	80	22	58	102	[59]	1
*S. marmoratus*	Friuli–Venezia	IT	57 ^†^	80	14/8	58	102	[55]	2; 5
*S. obtusirostris*	Buna R. (Neretva R. basin)	BIH	/	82	12	70	94	[60]	1
*S. peristericus*	Aigos Germanos	GR	/	80	20	60	100	[56]	1; 2
*S. trutta* *	Buni, Krupica, Bistrica R.	RU	17	80	18–20	62–60	100	[61]	1; 2
*S. trutta* *	Klinje L.	BIH	17	80	20	60	100	[61]	1; 2
*S. trutta* *	Pschata R.	SI	/	80	20	60	100	[58]	1
*S. trutta* *	Tripotamos R.	GR	/	76	16	60	92	[56]	1; 2
Baltic Sea Basin									
*S. trutta*	Ropsha	RU	/	78	20	58	98	[62]	3
*S. trutta (anadromous)*	Vistula R.	PL	23	80	14/6	60	100	[63]	
*S. trutta (anadromous)*	Vistula R.	PL	21	80	22	58	102	[64]	
*S. trutta*	Vistula R.	PL	18	80	22	58	102	[65]	
*S. trutta (lacustrine)*	Wdzydze L.	PL	13	80	22	58	102	[65]	
*S. trutta*	Gawrych Ruda Hatchery	PL	21	80	22	58	102	[66]	
Black Sea Basin									
*S. labrax*	Local hatchery	GE	embryos	80	18	62	98	[67]	1
*S. labrax*	Local hatchery	GE	6	80	22	58	102	[68]	
*S. trutta* *	Black R.	GE	8	80–82	20–22	60	100–104	[68]	3
*S. trutta* *	Bzyb R.	GE	9	82	22	60	104	[68]	1
*S. trutta* *	Gumista R.	GE	9	82	22	60	104	[68]	1
*S. trutta* *	Kodori R.	GE	8	80–82	20–22	60	100–104	[68]	1; 2
*S. trutta* *	Bicaz, Prejmer, Azuga	RO	/	80	24	56	104	[69]	1; 2
*S. trutta* *	Western–Middle Carpathians	RO	/	80	24	56	104	[69]	1; 2
Caspian Sea Basin									
*S. caspius*			embryos	80	18	62	98	[70]	1
*S. caspius*	Kura R.	AZ	2	82	20	62	102	[68]	1; 2
*S. ischchan “winter ischchan”*	Sevan L.	AR	11	80	16	64	96	[67,71]	1; 2
*S. ischchan “gegarkuni”*	Sevan L.	AR	17	80	18	62	98	[67,71]	1; 2
*S. ischchan “summer ischchan”*	Sevan L.	AR	23	82	18	64	100	[67,71]	1; 2
*S. ischchan “bodjak”*	Sevan L.	AR	7	82	16	66	98	[67,71]	1; 2
*S. trutta “alabalach”* *	Argichi R.	AR	8	80	16	64	96	[72]	1
*S. trutta*	Marmarik R.	AR	/	82	16	66	98	[73]	1
*S. trutta*	Vedi R.	AR	/	78	20	58	98	[73]	1
*S. trutta* *	Azat R.	AR	8	78	20	58	98	[68]	1
*S. trutta* *	Arindg R.		7	80	18	62	98	[68]	1
*S. trutta* *	Vedi R.	AR	18	78	20	58	98	[68]	1
*S. trutta* *	Korotan R.		15	80	20	60	100	[68]	1
*S. trutta* *	Dzeoraget R.	AR	8	80	20	60	100	[68]	1
*S. trutta* *	Kcia R.		3	82	20	62	102	[68]	1
*S. trutta* *	Kyuretchai R.		9	84	16	68	100	[68]	1
*S. trutta* *	Marmarik R.	AR	7	82	16	66	98	[68]	1
*S. trutta* *	Ochtchi R.		8	82	20	62	102	[68]	1
*S. trutta* *	Chatchen R.		7	80	20	60	100	[68]	1
*S. trutta* *	Tchaki R.		8	82	18	64	100	[68]	1
*S. trutta* *	Goygol L.	AZ	7	80	20	60	100	[68]	1
*S. trutta* *	Tabackuri L.	GE	15	80	20	60	100	[68]	1
Northern Sea Basin, European Atlantic coast									
*S. trutta*	Cares R.	ES	49	80	22–23	57–58	102–103	[74]	1
*S. trutta*	Pyrenees hatchery	ES	44	81	22–24	57–59	103–105	[74]	1
*S. trutta (anadromous)*	Galicia	ES	14	80	20	60	100	[75]	4
*S. trutta (local hatchery strain)*	Galicia	ES	19	80	20	60	100	[75]	4
*S. trutta*	Pšovka Cr.	CZ	10	80	14/4	62	98	[76]	4
*S. trutta*	Navia, Tambre, Umia, Mino R.	ES	133	78–80	20	58–60	98–100	[77,78]	2; 4; 5
*S. trutta*	Galicia	ES	15	80	20	60	100	[79]	3
*S. trutta*	Hatchery stock AT lineage	IT	20	80	14/8	58	102	[55]	2; 5
*S. trutta*	Loch Lomond	SCT	6	79–80	21–22	58–59	100–102	[80]	3
*S. trutta*	Norway (migratory)	NO	/	80	14	66	94	[81]	3
*S. trutta*	Germany	DE	6	78–82	20–26	52–62	102–104	[82]	2
*S. trutta*	10 localities across all Sweden	SW	14	80	20	60	100	[83]	1

Notes: 1: Giemsa-stained chromosomes only; 2: Robertsonian polymorphism detected; 3: Replication banding pattern discovered cytotype variants in some chromosomes; 4: Ag-, CMA_3_- and/or C-banding, cytotype polymorphisms; 5: Ag-, CMA_3_- and rDNA ISH and/or FISH; *—Material was analysed under the name *S. trutta* but evidently out of the known autochthonous range of *S. trutta s. str*., thus likely representing another species of the genus, the species name was determined based on locality data in a given study according to geographical distribution of trout taxa in Kottelat and Freyhof [4]; †—counts reported both trout taxa without distinguishing between them. Studies with incomplete information (without data reflecting karyotype composition, geographic origin, number of examined individuals or methodically problematic studies; all mostly reviewed in Gas [49]) were excluded from this review; symbol “-“ in chromosome counts represents observed range, symbol “/”in chromosome counts shows that both categories (m and sm or st and a) were determined.

The karyotype of flathead trout undoubtedly belongs to category A sensu Phillips and Ráb [18], i.e., salmonids with 2n = ~80 and chromosome arm number NF = ~100. The 2n = 80 found in flathead trout has been reported in a majority of studies (e.g., [16,37,48]). Differences from this value are mostly caused by centric fusions of acrocentric chromosomes and/or fissions of metacentric chromosomes as reported in nearly all studies so far. Some reports of different 2n were caused by (i) lower quality of metaphases examined and/or (ii) low number of analysed individuals (e.g., Kaidanova [62], Karakousis et al. [56]). On the other hand, some studies pointed definitively to different 2n such as 2n = 82 in S. obtusirostris [60], 2n = 84 in S. trutta “alabalach” [72], 2n = 82 in some forms of S. ischchan [71] and 2n = 78 to 82 in some taxonomically unidentified Transcaucasian trout [68]. Regardless, such variation in 2n, frequently documented in other lineages of salmonids with A type karyotype, could be explained despite minor chromosome rearrangements such as pericentromeric inversions that can convert acrocentric chromosomes into sub-telocentric ones [18].

The 2n, karyotypes, and hence NF of the examined species/forms of brown trout are remarkably similar (Table 1). Nevertheless, differences caused by a chromosome classification bias among individual reports exist. Most authors categorise uni-armed and bi-armed chromosomes according to Levan [38] but NF was originally designed to quantify the centric translocations or fissions of the Robertsonian type [39] only. However, some authors scored sub-telocentric chromosomes as bi-armed. Differences in the NF reported for the same form/species thus usually resulted from a difference in the scoring rather than from any real variation. In other words, most of the studies provide the number of metacentric and submetacentric chromosomes together, while a minority of them distinguish these categories, as was done in our study. Another problem in comparing reports on karyotype structures in brown trout is that most of the summarized studies analysed Giemsa-stained chromosomes, published karyotypes and/or metaphase plates of lower quality to infer karyotype structure in more details. The studies using conventional and/or molecular cytogenetic protocols [55,64,65,66,74,75,76,77,78,79,80] revealed very similar or even identical karyotypes as we found in the flathead trout for this study. We can therefore conclude that the karyotype of brown trout typically consist of seven pairs of metacentric, five to six pairs of visibly sub-telocentric chromosomes and all remaining are acrocentric elements of gradually decreasing size. The brown trout’s karyotype also contains several distinct chromosome markers—the first two pairs of acrocentric chromosomes distinctly larger from other acrocentric ones and the largest sub-telocentric pair, which is also the largest one in the complement. The short (p) arm of this marker chromosome pair bears the major rDNA sites, as revealed by FISH with 28S rDNA probe, corresponding to positive Ag- and CMA_3_-stainings [55,64,65,66,75,76,77,78,84]. Intraspecific variation in the locations and sizes of the chromosomal nucleolar organizer regions (NORs), i.e., major rDNA sites, have been frequently documented [85] but available data for this marker consistently document the same karyotype location across brown trout diversity including flathead trout. However, as in other cases, some intraspecific variability has been observed [64,65,66]. In our study, we observed the variability in the size of the NOR-bearing p arm of this marker chromosome corresponding to the 28S rDNA signal, similar to Caputo et al. [28] in S. marmoratus. The intraspecific variability of the 5S rDNA cytotaxonomic marker is quite well known [85,86,87]. Surprisingly, the location of 5S rDNA genes in the genus Salmo was examined in two studies only. Pendás et al. [84] found multichromosomal sites of these genes in brown trout from northwestern Spain, while Caputo et al. [55] observed these sites in telomeres of one middle-sized metacentric pair only. Our study also detected this gene cluster in the pericentromeric region of one middle-sized metacentric pair only. Whether this 5S rDNA bearing chromosome pair is homologous remains to be demonstrated by a cross-species painting protocol (e.g., Ráb et al. [88]). We can therefore conclude that the 2n, and structure as well as number and position of NORs, i.e., the active 28S rDNA sites, of the endemic flathead trout karyotype entirely correspond to those found in other brown trout taxa

### 4.1. Cytotaxonomy and Diversity of Eurasian Trouts

The species *S. trutta* has long been considered a single but highly polymorphic species broadly distributed in the European ichthyo-geographic region (see Bañarescu [42]) forming three ecotypes—marine migratory, lacustrine and brook/riverine [4]. In line with this, several subspecies or even distinct species have been described but most of them are simply considered as interindividual and/or interpopulation variability. Though even nominal subgenera of the genus *Salmo* have been described, i.e., *Acantholingua* (for *A. ohridanus*), *Salmothymus* (for *S. obtusirostris*) and *Platysalmo* (for *P. platycephalus*), collectively called ´archaic trout´, they are closely related to the *S. trutta* species complex at the molecular level (e.g., Sušnik et al. [89], Phillips et al. [90]). However, recent detailed investigations of brown trout life histories, biology, distribution and taxonomy suggest that the biological and hence taxonomic diversity of the Eurasian genus *Salmo* is considerably greater than the taxonomy that was accepted up until the 1990s would suggest [4,91], a situation similar for freshwater trout of the genus *Oncorhynchus* [92]. Recently, FishBase [93] lists 50 formally described *Salmo* species. However, many molecular phylogeneticists and phylogeographers question this biological species concept of taxonomic diversity of the genus *Salmo* by pointing to negligible and/or weak genetic differentiation among some of those populations/taxa (to cite from numerous ones e.g., [13,89,94,95,96,97,98,99]). Yet, other colleagues detected significantly larger genetic differences (e.g., [100,101,102,103,104]). How can the cytotaxonomy of the genus *Salmo* contribute to this debate? Our results of the cytogenetic analysis of flathead trout compared with available cytogenetic data for other trout populations and/or taxa (Table 1) clearly demonstrate that 2n, karyotype structures and other chromosomal markers, especially the position of major rDNA sites, are rather stable or even invariable across trout diversity as described for several lineages of salmonid fishes with A type karyotypes [18,105]. At first glance, this conclusion would support/conform to the view of molecular-based studies. However, the stability of 2n and similar and/or even identical chromosomal characteristics were observed, i.e., karyotype stasis is widely documented in a group of taxonomically different species and/or even lineages. Such uniform stasis has been discovered in groups as diverse as plants [106,107,108], amphibians [109,110] and birds [111]. Among teleost fishes, multiple groups display such apparent karyotype stasis persisting in significantly long stages of lineage divergences, e.g., pikes of the genus *Esox* [112,113], fishes of the family Leuciscidae ([88,114,115,116,117] and references therein), Gobionidae [34], Xenocyprinidae [118] and especially many percomorph groups [119,120,121,122,123,124]. The underlying evolutionary mechanisms for this mode of karyotype (non) differentiation have not been identified so far but they may be at least partially linked with the functional arrangement of chromatin within the interphase nucleus and the degree of tolerance to its change [125,126]. We therefore conclude that, from the cytotaxonomic point of view, apparent karyotype stasis found in trout of the genus *Salmo* does not challenge their existing and evident taxonomic diversity.

### 4.2. Cytogenomics in Salmonids

The cytogenomic approach represents a logical continuation of the traditional molecular cytogenetics, which was crucial for understanding fish genome evolution. Cytogenomics effectively integrates the huge body of evidence generated by karyological and cytogenetic research with the genomic approach based on currently extensive genome sequencing [127]. The sequencing effort of fish genomes is still accelerating and highly ambitious; hence, with about 32,000 fish species [128], fish cytogenomics has a good chance of fast becoming as equally crucial as molecular cytogenetics despite the small fraction of genomes that had been sequenced so far in comparison with the number of species already analysed cytogenetically. Already, virtual karyotyping has taken another step forward with the potential to visualize more details with better resolutions than through the use of microscopes for most small-sized fish chromosomes.

At this initial stage, our tool for virtual karyotyping utilizes masking of repeats in the DNA sequence via soft-masking, i.e., identified repetitive sequences become lower-case, whereas the remaining sequences retain their upper-case. It means that the quality of the input assembly and its soft-masking is crucial and cannot be influenced by the tool itself. This tool has been introduced in this special issue to outline the potential future of fish cytogenomics, and so far its functionality has been utilized to address general questions on GC% and repeats evolution not only in fish but also across vertebrates. This means that the tool has not yet been used systematically in cytogenetically analysed fish species and results of both approaches have not yet been compared.

The inverse relationship between the GC% and genome size had been initially ascribed to the extremely dynamic and often highly amplified ribosomal genes [105,129,130,131] that represent the GC-richest genome fraction [132,133]. However, further molecular cytogenetic results based on FISH with rDNA probes in further salmonids (continuously summarized by the database by Sochorová et al. [134]) as well as the results obtained here do not support these initial assumptions. The genomic approach is less useful here, because the rDNA is mostly disregarded and/or even discarded in the genome assemblies.
